# Supplementary material for: Systematic Evaluation of Antigenic Stimulation in Chronic Lymphocytic Leukemia: Humoral Immunity as Biomarkers for Disease Evolution
Source: Cancers (Basel). 2023 Jan 31;15(3):891. doi: 10.3390/cancers15030891 (PMC9913429; doi:10.3390/cancers15030891)
Supplement: Supplementary file 1 [file cancers-15-00891-s001.zip › cancers-2057018-supplementary/Supplementary Figures/Supplementary Figures.pdf]

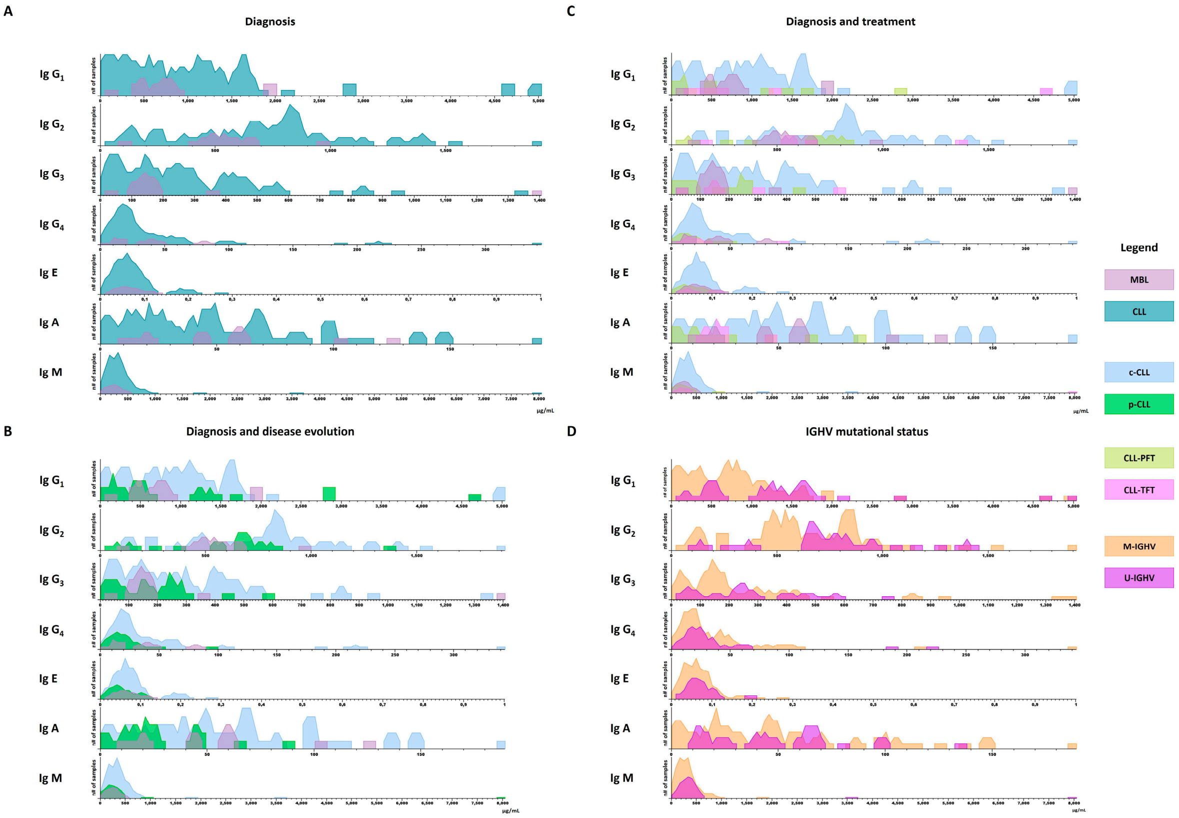

Figure S1. Distributions of the immunoglobulins' quantitative levels according to clinical/diagnosis subsets: (**A**) MBL and CLL groups; (**B**) MBL, c-CLL, and p-CLL groups; (**C**) MBL, c-CLL, and treatment (CLL-PFT and CLL-TFT) groups; (**D**) IGHV mutational status (M-IGHV or U-IGHV) groups.

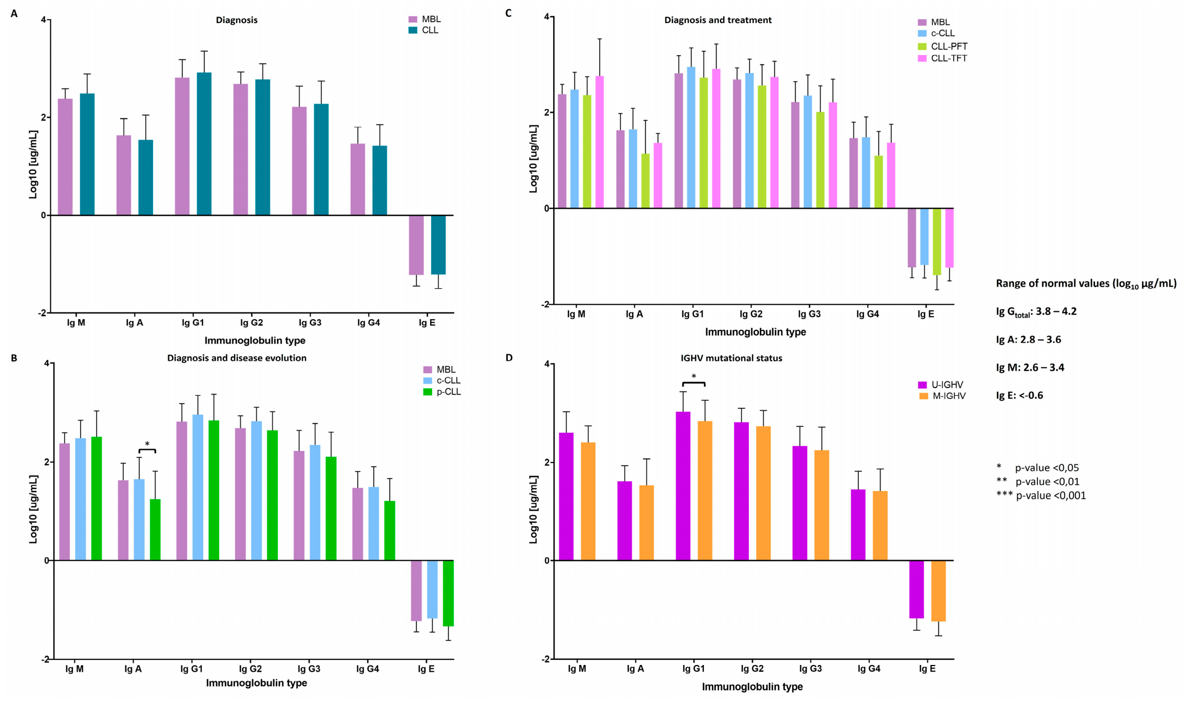

Figure S2. Quantitative distribution profiles for each immunoglobulin according to patient subsets: (**A**) diagnosis groups; (**B**) diagnosis and disease evolution groups; (**C**) diagnosis and treatment groups; (**D**) IGHV mutational status groups.

.

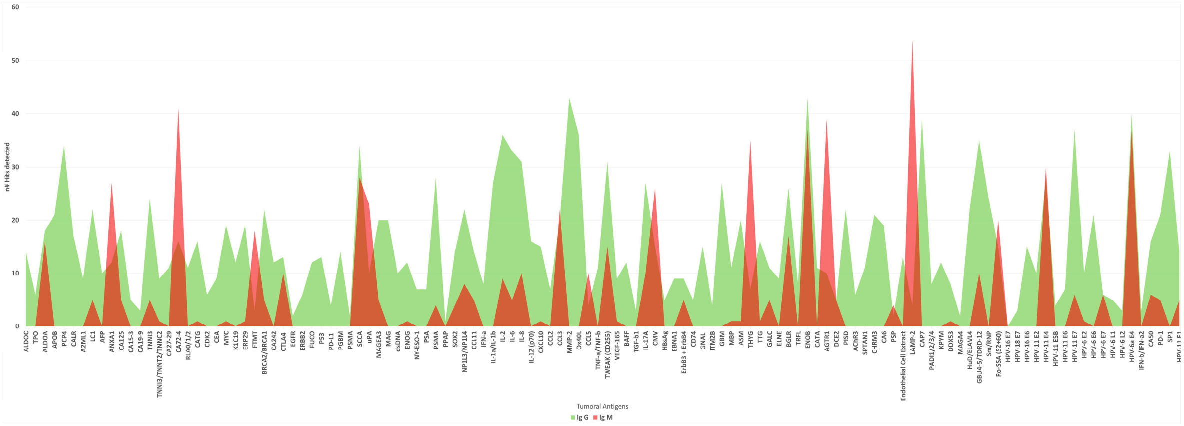

Figure S3. Distribution of tumoral antigens (TAs) detected in this study according to presence of Ig G or Ig M autoantibodies.

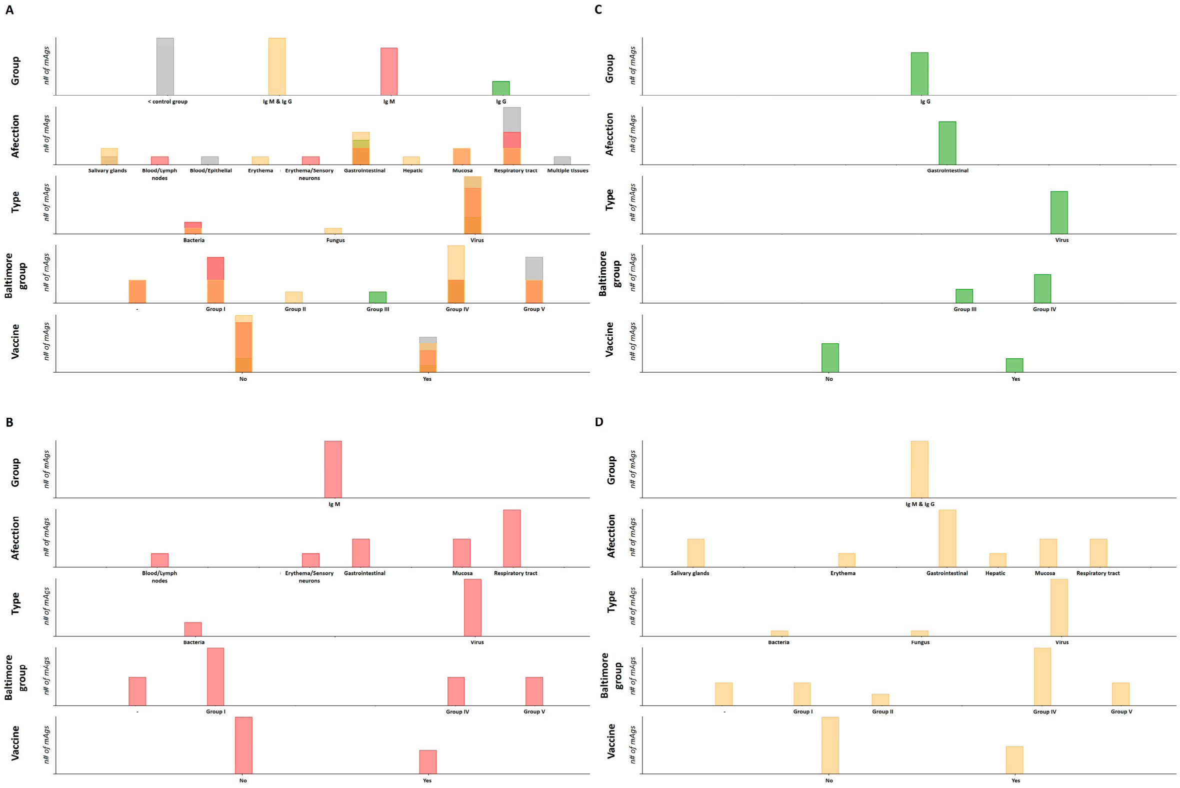

Figure S4. Summary of antimicrobial antibodies (Ig G and Ig M) detected at 0.0001 μg/μL [antigen]: (**A**) distribution according to immunoglobulin isotype detected in the studied cohort; (**B**) distribution of antimicrobial Ig M; (**C**) distribution of antimicrobial Ig G; (**D**) distribution of antimicrobial Ig M and Ig G).

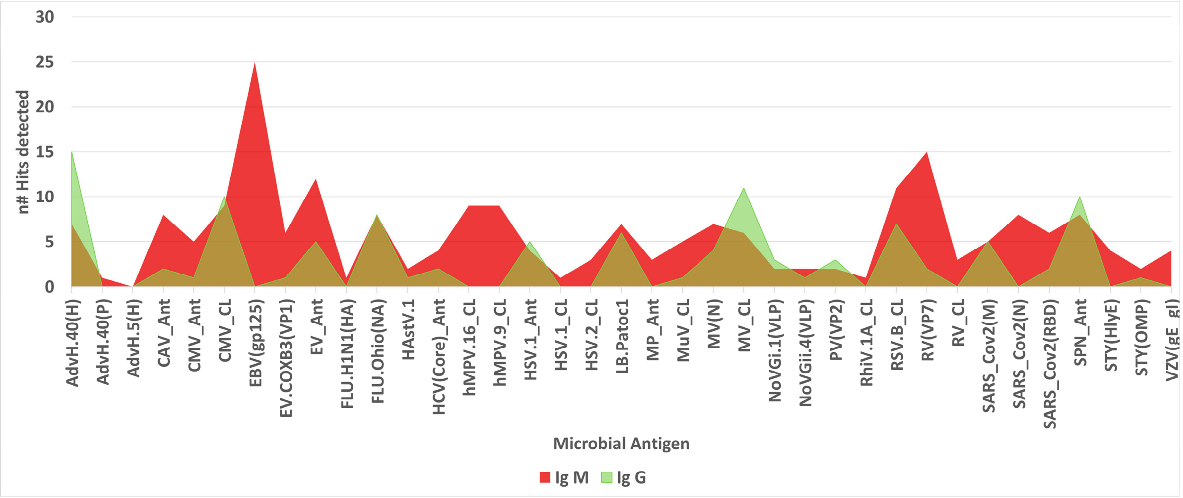

Figure S5. Quantitative summary of microbial antigens detected in this study.
