# Supplementary figures and images for: Systematic Evaluation of Antigenic Stimulation in Chronic Lymphocytic Leukemia: Humoral Immunity as Biomarkers for Disease Evolution
Source: Cancers (Basel). 2023 Jan 31;15(3):891. doi: 10.3390/cancers15030891 (PMC9913429; doi:10.3390/cancers15030891)

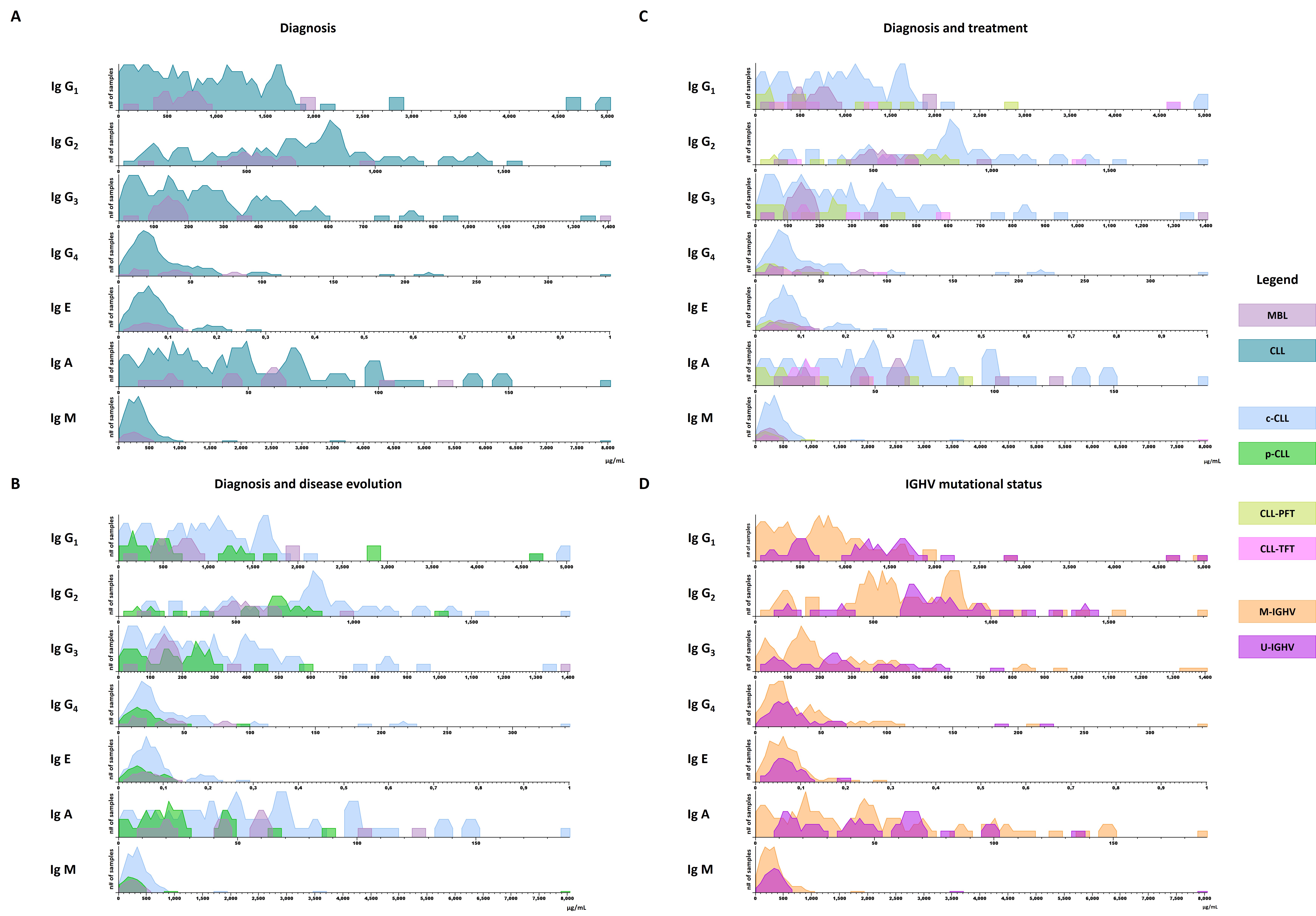

Supplement: Supplementary file 1 [file cancers-15-00891-s001.zip › cancers-2057018-supplementary/Supplementary Figures/Figure S1.jpg]

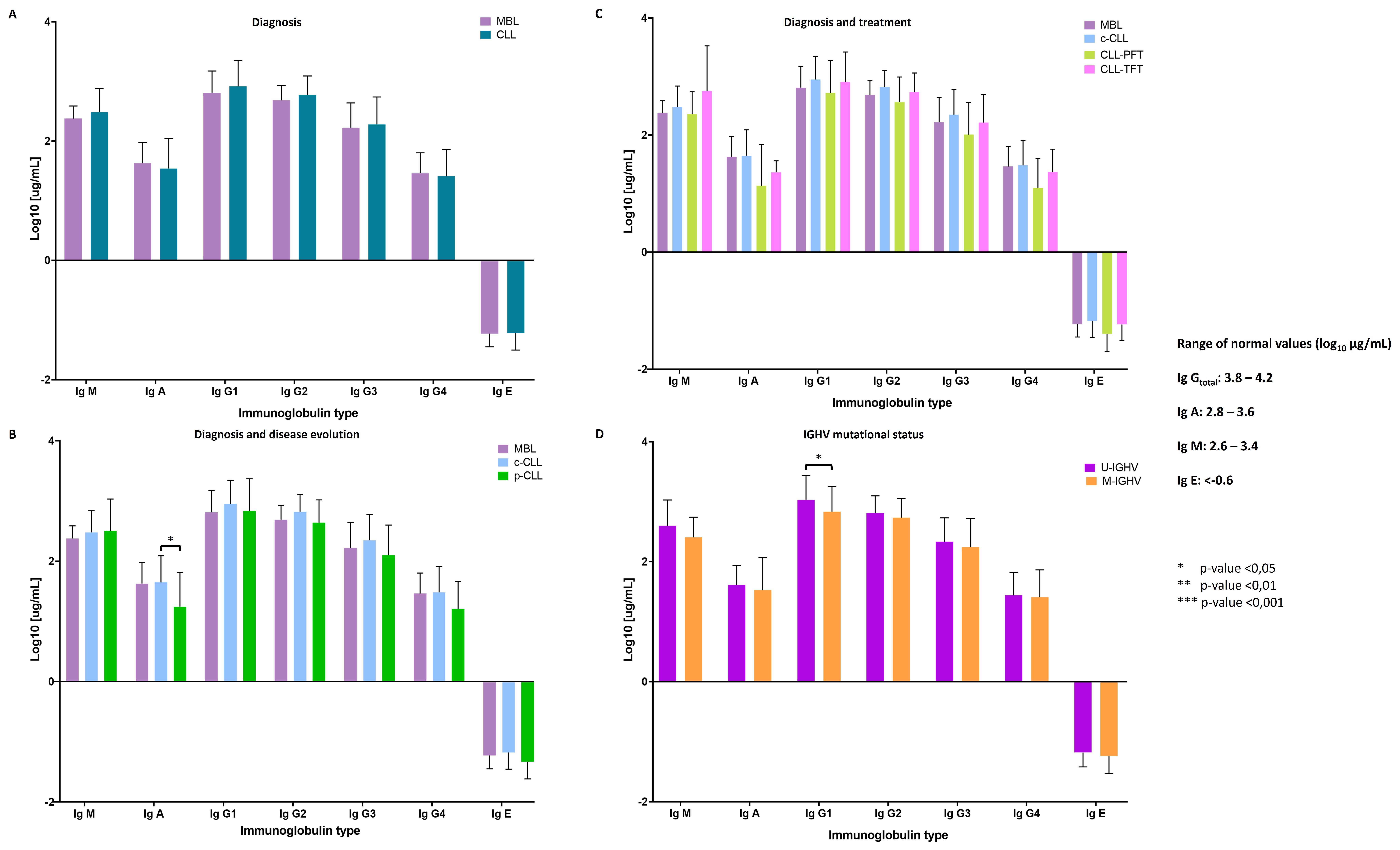

Supplement: Supplementary file 1 [file cancers-15-00891-s001.zip › cancers-2057018-supplementary/Supplementary Figures/Figure S2.jpg]

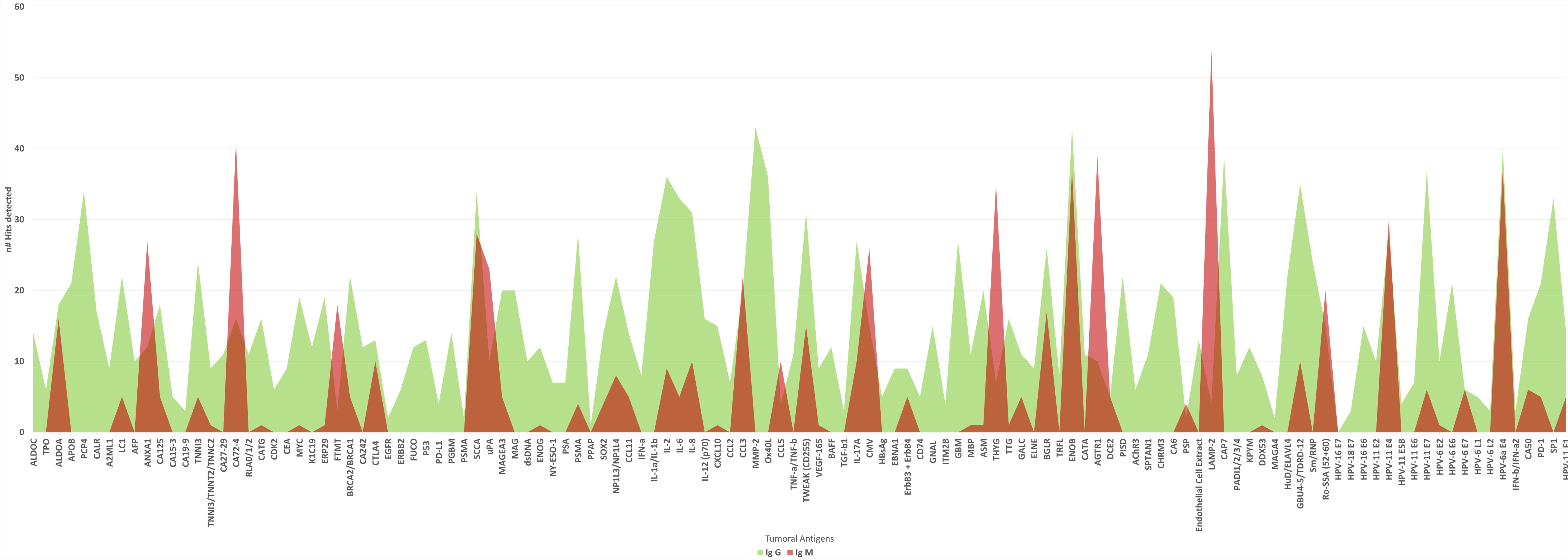

Supplement: Supplementary file 1 [file cancers-15-00891-s001.zip › cancers-2057018-supplementary/Supplementary Figures/Figure S3.jpg]

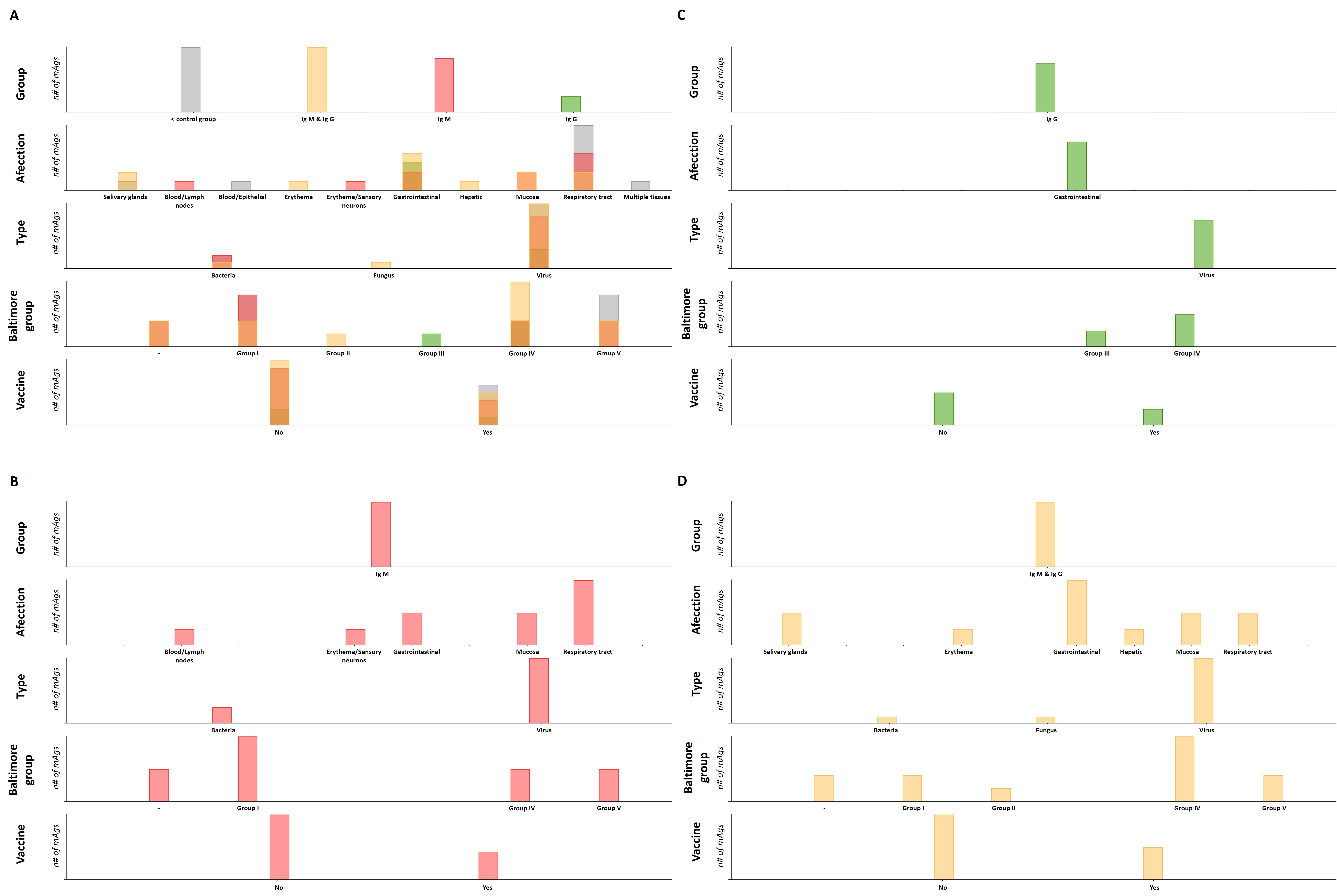

Supplement: Supplementary file 1 [file cancers-15-00891-s001.zip › cancers-2057018-supplementary/Supplementary Figures/Figure S4.jpg]

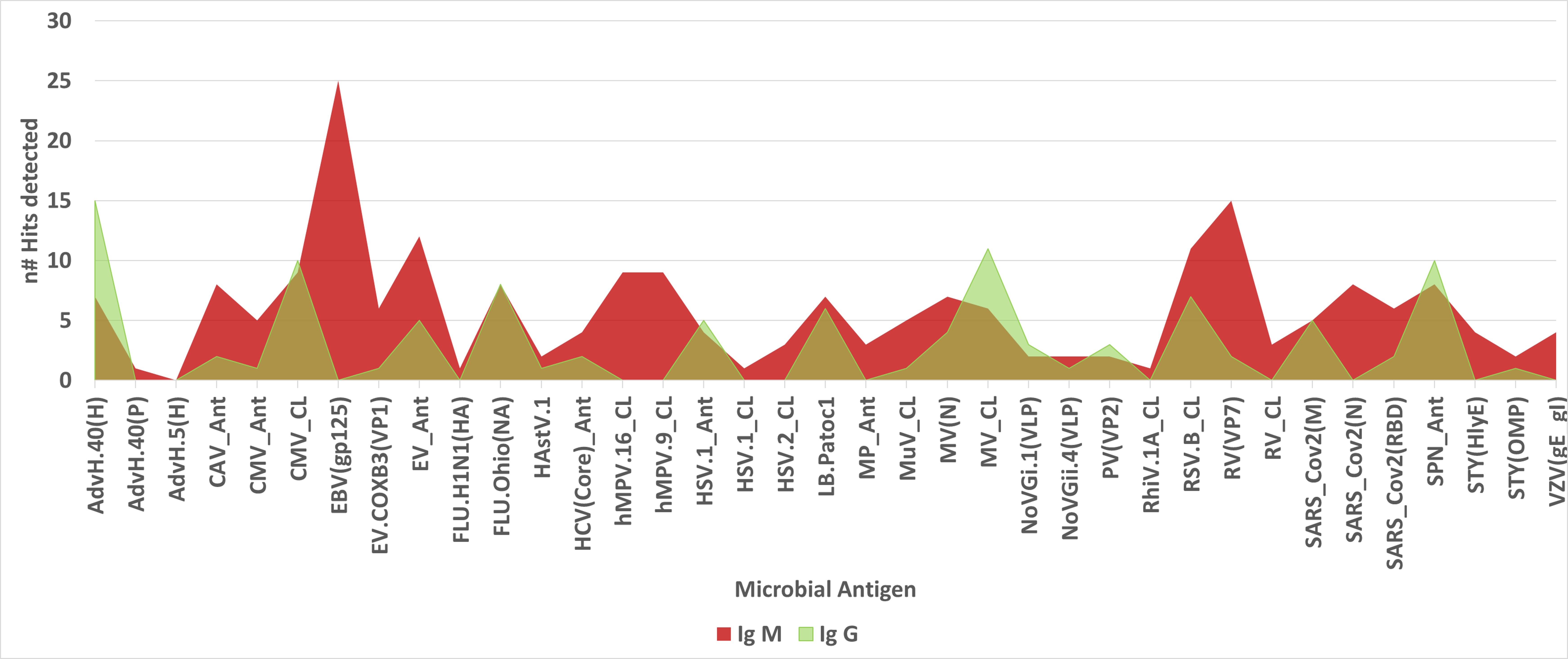

Supplement: Supplementary file 1 [file cancers-15-00891-s001.zip › cancers-2057018-supplementary/Supplementary Figures/Figure S5.jpg]
